# Rhodium Porphyrin Bound to a Merrifield Resin as Heterogeneous Catalyst for the Cyclopropanation Reaction of Olefins

**DOI:** 10.3390/molecules21030278

**Published:** 2016-02-27

**Authors:** Alina Ciammaichella, Valeria Cardoni, Alessandro Leoni, Pietro Tagliatesta

**Affiliations:** Dipartimento di Scienze e Tecnologie Chimiche, University of Rome-Tor Vergata, Via della Ricerca Scientifica, 00133 Rome, Italy; alina07@hotmail.it (A.C.); valery881@virgilio.it (V.C.); alessandro.leoni@uniroma2.it (A.L.)

**Keywords:** porphyrin catalysts, cyclopropanation reactions, immobilized catalysts

## Abstract

Cyclopropanation reaction is an important tool for obtaining interesting compounds and can be catalyzed by metalloporphyrins with high *syn/anti* ratio. The catalyst cannot be recycled and is usually lost during chromatographic separation from the two isomeric products. In this paper a *meso*-tetraphenylporphyrin rhodium(III) chloride was bound to a Merrifield resin and used to catalyze the cyclopropanation reaction of nine olefins, giving good yields and selectivities of the final products and for the first time, a partial recycling of the catalyst. This new catalytic system will be tested in the future for the synthesis of natural products containing cyclopropyl ring.

## 1. Introduction

The cyclopropyl ring is an important organic function due to the presence of such structure in a number of interesting natural products. Recently, this ring has been found in molecules with antileukemic activity *in vitro* [1]. Several methods have been discovered in the past for obtaining such ring using copper, rhodium and osmium complexes as efficient catalysts for the synthesis of cyclopropanes from diazocompounds [2]. Synthetic iron, rhodium and osmium porphyrins have been also reported as catalysts for the cyclopropanation reaction of simple olefins by ethyldiazoacetate (EDA) [3,4,5,6,7,8,9]. Compared with the copper catalysts, like CuCl which preferentially affords the *anti* isomers, the porphyrin catalysts give interesting results in reversing the *syn/anti* ratio of the products depending on the nature of the metal. The reaction mechanism of the metalloporphyrins catalyzed cyclopropanation reactions is not completely elucidated in all the aspects, because of the lability of the bond between the central metal and the acetate residue.

**Scheme 1:**
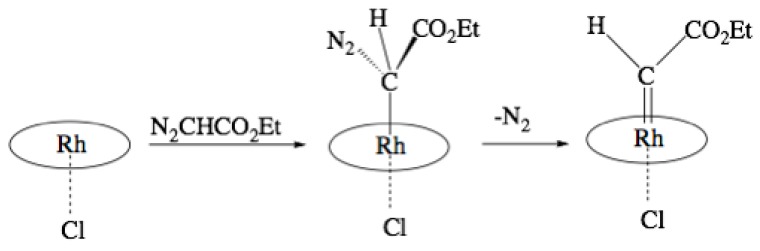
Carbene formation mechanism.

However, the intermediate of the reaction, shown in Scheme 1, proposed, in the case of rhodium, by Callot *et al.* [10] was later studied by Kodadek, who used the NMR spectroscopy for detecting the possible carbene species [4].

During our studies on the catalytic properties of metalloporphyrins, we found that rhodium or ruthenium complexes of such macrocycles are able to catalyze the cyclooligomerization of arylethynes to give dimers or trimers, depending from the reaction conditions and substrates [11,12,13,14,15]. We also showed the possibility to bind a ruthenium porphyrin to the Merrifield resin to give an heterogeneous system able to catalyze the cyclooligomerization of alkynes with a complete recovery of the catalyst by simple filtration [16].

The recovering of the catalyst without any tedious column separation, is an important goal in preparative chemistry because this fact allows us to synthesize large amount of the products using small quantity of expensive metal complexes. In this paper we will show the possibility to use an immobilized metalloporphyrin as catalyst for the cyclopropanation of standard olefins, with good yields in some cases and high *syn/anti* ratios comparable with those obtained using normal metalloporphyrins in homogeneous solution [10].

## 2. Results

**Figure 1:**
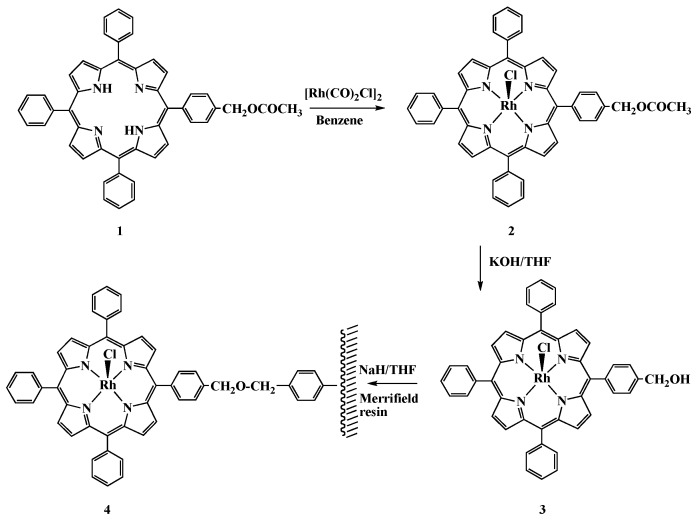
Synthetic pathway for obtaining the catalyst **4**.

The heterogeneous catalyst was obtained by using the well known Merrifield technique [17] which was applied for the first synthesis of peptides in solid phase. The starting porphyrin free base, **1** was synthesized as reported in the literature [18] and rhodium complex **2** was obtained by a standard method [19]. After saponification with KOH, the rhodium porphyrin **3** was bound to the solid resin using the Williamson method for the synthesis of ethers [20]. The synthetic steps are reported in Figure 1.

From the data of the elemental analysis obtained for the catalyst **4**, we estimate the content of the rhodium porphyrin to be around 1 mmolar per gram of solid, a value very close to those obtained for other porphyrin functionalized solids [21,22,23,24]. The binding of the catalyst **3** to the solid through the ethereal function allows to separate the functionalized resin from the bulk of the reaction by vacuum filtration and reuse it.

Furthermore, the robustness of the system at the reaction temperature (65 °C) was also verified by UV/vis methods. In fact after 48 h of reaction at this temperature no trace of free metalloporphyrin was detected in the reaction media by UV/vis spectra. In Table 1 we report the data obtained using nine simple olefins as substrates in the cyclopropanation reaction catalyzed by **4**. The catalytic activity of the rhodium porphyrin bound to the resin is compared with that obtained from literature (in parentheses) for styrene, cyclohexene and norbornene.

In our opinion these results support the fact that there is no influence of the polymeric matrix on the yields and *syn/anti* ratios.

## 3. Discussion

From the data it is clear that the yields of the reactions are slightly lower whilst the *syn/anti* ratios are comparable with those obtained using rhodium porphyrins in homogeneous solution. The turnover numbers are between 10^3^ and 10^4^ for all the reactions, much higher than those previously reported [10].

Only when cyclohexene is used as substrate the *syn/anti* ratio increases. The recycle of the catalyst did not give good results in the case of styrene which has been used as standard substrate. In fact the yield decreases to 42% in the second run but this value remains almost constant for the third and fourth run, showing that the catalyst retains part of its activity. It should be noted that to our knowledge, this is the first example of the reuse of a catalyst for the cyclopropanation reaction. Furthermore, the lower yield reported in the text for the reuse of the catalyst **4** after the first run, seems to be not much affected by the temperature. In fact a yield of 50% for the reaction on styrene using **4** after a thermal treatment before the use, is almost comparable to that reported in Table 1, entry 1. Control experiments were performed using catalyst **2** and **3** in homogeneous solution and **4** after treatment at 65 °C in chloroform for 6 h, using styrene as standard olefin. In Table 2 the results of such reactions are reported. In Supplementary Materials, experimental procedure and characterization of new compounds are reported.

Both yield and *syn/anti* ratio for entries 1 and 2 are comparable with those obtained with catalyst **4** in the same experimental conditions (see Table 1, entry 1).

## 4. Materials and Methods

### 4.1. General

UV/vis spectra were recorded with a Varian Cary 10 spectrophotometer (Palo Alto, CA, USA). ^1^H-NMR spectra were recorded on a Bruker AM 400 spectrometer (Billerica, MA, USA) as CDCl_3_ solutions. Chemical shifts are given in ppm from tetramethylsilane (TMS) and are referenced against residual solvent signals. Mass spectra (FAB) were recorded on a VG-Quattro spectrometer (Fisons, Manchester, UK) using 3-nitrobenzylalcohol (NBA) as a matrix. The products yield and the isomeric ratios for all the reactions were determined by GC analyses (Waltham, MA, USA) performed on a Focus Thermo instrument equipped with a 15 m Restek MTX-5 capillary column and a FID detector. Chemical yields were determined by adding a suitable internal standard (decane or dodecane) to the reaction mixture at the end of each experiment and were reproducible within ±2% for multiple experiments.

### 4.2. Chemicals

All the reagents and solvents (Aldrich, St. Louis, MO, USA) were of the highest analytical grade and used without further purification. 

Silica gel 60 (70–230 and 230–400 mesh, Merck, Darmstadt, Germany) was used for column chromatography. High-purity grade nitrogen gas was purchased from Rivoira. 

The free base 5-(4-acetoxymethylphenyl)-10,15,20-triphenylporphyrin was synthesized by literature methods [18]. 

Rhodium(III) *meso*-tetraphenylporphyrin chloride was obtained as reported in the literature. [19] Analytical data for cyclopropane derivatives of 4-chloro, 4-methyl and 4-methoxystyrene are reported in the literature [25].

## 5. Conclusions

In synthesis we have prepared a new heterogeneous catalytic system using a rhodium porphyrin immobilized on the Merrifield resin and used it for the cyclopropanation of nine standard olefins. The catalyst can be separated from the bulk of the reaction by a simple filtration and retains part of its catalytic activity. New studies on the use of similar strategies for obtaining heterogeneous porphyrin catalysts for other reactions are currently in progress.

## Figures and Tables

**Table 1:** Cyclopropanation of olefins catalyzed by **4** as catalyst compared with the data from previous papers.

Entry	Substrate	Yield (%) ^b^	*syn/anti* Ratio ^c^	*syn/anti* Ratio from Lit. ^d^
1	Styrene ^a^	60(71 ^d^)	1.12	1.13
2	4-chlorostyrene	55	1.10	-
3	4-methylstyrene	53	1.30	-
4	4-methoxystyrene	59	1.85	-
5	Cyclohexene ^b^	48(62 ^d^)	1.03	0.84
6	Norbonene ^b^	51(71 ^d^)	1.27	1.28
7	1-Methylcyclopentene ^b^	45 ^e^	0.9 ^e^	-
8	2,4,4-Trimethylpentene ^a^	70 ^e^	0.72 ^e^	-
9	*trans*-β-Methylstyrene ^b^	52	0.15	-

^a^ Reactions carried out at 65 °C in CHCl_3_. ^b^ Reactions carried out at 45 °C in CH_2_Cl_2_ for 12 h with 3.7 mmol of substrate and 20 mg of catalyst **4**. ^c^ Yields determined by GC analysis. ^d^ From ref. 10 with Rh(III)(TPP)I as catalyst. ^e^ This paper.

**Table 2:** Cyclopropanation of styrene catalyzed by **2**, **3** and **4**.

Entry	Catalyst	Yield(%) ^a,b^	*syn/anti* Ratio
1	**2**	59	1.04
2	**3**	58	0.92
3	**4 ^c^**	50	1.06

^a^ Reactions carried out at 65 °C in CHCl_3_. ^b^ Yields determined by GC analysis. ^c^ After treatment of the catalyst at 65 °C in chloroform for 6 h.

## Supplementary Materials

Supplementary materials can be accessed at: http://www.mdpi.com/1420-3049/21/3/278/s1.
