# Supplementary material for: Rhodium Porphyrin Bound to a Merrifield Resin as Heterogeneous Catalyst for the Cyclopropanation Reaction of Olefins
Source: Molecules. 2016 Feb 27;21(3):278. doi: 10.3390/molecules21030278 (PMC6272877; doi:10.3390/molecules21030278)
Supplement: Supplementary file 1 [file molecules-21-00278-s001.pdf]

Supplementary Materials: Rhodium Porphyrin Bound to a Merrifield Resin as Heterogeneous Catalyst for the Cyclopropanation Reaction of Olefins

Alina Ciammaichella, Valeria Cardoni, Alessandro Leoni and Pietro Tagliatesta

Instrumentation

UV-vis spectra were recorded with a Varian Cary 10 spectrophotometer. ^{1}H-NMR spectra were recorded on a Bruker AM 400 spectrometer as $CDCl_3$ solutions. Chemical shifts are given in ppm from tetramethylsilane (TMS) and are referenced against residual solvent signals. Mass spectra (FAB) were recorded on a VG-Quattro spectrometer using 3-nitrobenzylalcohol (NBA) as a matrix.

GC Analysis

The products yield and the isomeric ratios for all the reactions were determined by GC analyses performed on a Focus Thermo instrument equipped with a 15 m Restek MTX-5 capillary column and a FID detector. Chemical yields were determined by adding a suitable internal standard (decane or dodecane) to the reaction mixture at the end of each experiment and were reproducible within ±2% for multiple experiments.

Chemicals

All the reagents and solvents (Aldrich) were of the highest analytical grade and used without further purification.

Silica gel 60 (70–230 and 230–400 mesh, Merck) was used for column chromatography. High-purity-grade nitrogen gas was purchased from Rivoira. The free base 5-(4′-acetoxymethylphenyl)-10,15,20-triphenylporphyrin was synthesized by literature methods.[17]

Rhodium(III) *meso*-tetraphenylporphyrin chloride was obtained as reported in the literature [18].

Synthesis

[5-(4-Acetoxymethylphenyl)-10,15,20-triphenylporphyrin]Rh(III)Cl (**2**). 100 mg of 5-(4-acetoxymethylphenyl)-10,15,20-triphenylporphyrin (**1**) (0.145 mmol) were dissolved in benzene (100 mL). 100 mg of $Rh[(CO)_2Cl]_2$, (0.156 mmol), were added and the solution was refluxed for 24 h in air. The solution was then cooled and evaporated under vacuum. The resulting solid was dissolved in a small quantity of chloroform and chromatographed on silica gel eluting with chloroform/hexane, 70:30. The fraction containing the rhodium complex was vacuum evaporated and the residue recrystallized from CH_2Cl_2/hexane (5:1). Yield 70%. Anal. Calcd for $C_{47}H_{32}N_4RhO_2Cl$: C, 68.57; H, 3.91; N, 6.80; Found: C, 68.67; H, 3.81; N, 6.76. ^{1}H-NMR (400 MHz, $CDCl_3$) δ = 8.89 (s, 8H), 8.15–8.25 (m, 8H), 7.60–7.73 (m, 11H), 5.47 (s, 2H), 2.25 (s, 3H);UV-vis ($CHCl_3$): 413, 520 nm. Low resolution mass spectrum (FAB) *m*/*z* 787 $[(TPP)Rh(CH_2OCOCH_3)]^+$.

[5-(4-Hydroxymethylphenyl)-10,15,20-triphenylporphyrin]Rh(III)Cl (**3**). 100 mg of [5-(4-acetoxymethylphenyl)-10,15,20-triphenylporphyrin]Rh(III)Cl were dissolved in 100 mL of THF under nitrogen and 5 mL of 1M solution of KOH in methanol were added. The solution was stirred at R.T. for 6 hours, then poured in 500 mL of water and extracted with three portions of 100 mL each of chloroform. The organic solution was then washed with water two times and dried on anhydrous Na_2SO_4. The solvent was evaporated under vacuum to a small volume and hexane was added to precipitate the product. Yield 95%. Anal. Calcd for $C_{45}H_{30}ON_4RhCl$: C, 72.48; H, 4.05; N, 7.51; Found: C, 72.41; H, 4.16; N, 7.55. ^{1}H-NMR (400 MHz, $CDCl_3$) δ = 8.91 (s, 4H), 8.75 (s, 2H), 8.70 (s, 2H), 8.20–826 (m, 7H), 7.70–7.82 (m, 12H) 5.07 (s, 2H), UV-vis (CH_2Cl_2): 412, 520 nm. Low resolution mass spectrum (FAB) *m*/*z* 745 $[(TPP)Rh(CH_2OH)]^+$.

Rhodium porphyrin-Merrifield resin adduct (**4**). 100 mg, (0.10 mmol) of [5-(4-hydroxymethylphenyl)-10,15,20-triphenylporphyrin]Rh(III)Cl, (**3**) were dissolved in 50 mL of anhydrous THF under nitrogen in a 100 mL flask. NaH, 46 mg (0.57 mmol), 80% oil slurry, were added and the mixture was refluxed for 1 h. The solution was cooled to room temperature and 200 mg of, 5% chlorine content, Merrifield resin were added. The mixture was again refluxed for 12 h, after that the solution was cooled and water, 1 mL, was carefully added. The resulting red solid was filtered and the filtrate washed with water and then with chloroform untill the solvent was uncoloured. The red solid was dried under vacuum at 60 °C for 3 h.

Syn and anti 1-ethoxycarbonyl-2-(2,2-dimethylpropyl)-2-methylcyclopropane (**5** + **6**). Ethyldiazoacetate, 1.1 mL (13.4 mmol) was added to a solution of 3.9 mL of 2,2,4-trimethylpentene(34.8 mmol) in 15 mL of dry CH_2Cl_2. TPPRh(III)Cl. 20 mg (26 mmol) was added and the solution was refluxed for 24 h under nitrogen. The solution was then evaporated under vacuum and the residue chromatographed on silica gel eluting with hexane/diethyl ether, 99.7:0.3.

The first eluted fraction contained 380 mg of the *anti* isomer (yield 19 %) while the second fraction contained 384 mg of the *syn* isomer (yield 20 %).

Syn isomer:Anal. Calcd for $C_{12}H_{22}O_2$: C, 72.68; H, 11.18; Found: C, 72.65; H, 10.91; ^{1}H-NMR (300 MHz, $CDCl_3$) δ = 4.11 (q, *J* = 8 Hz, 2H), 3.42 (t, *J* = 8 Hz, 3H), 1.45 (s, 3H), 1.36–1.41 (m, 1H), 1.13–1.7 (m, 7H), 094 (s, 9H); GC-MS *m*/*z* 199 $[M + H]^+$.

Anti isomer:Anal. Calcd for $C_{12}H_{22}O_2$: C, 72.68; H, 11.18; Found: C, 72.70; H, 11.02; ^{1}H NMR (300 MHz, $CDCl_3$) δ = 4.12 (q, *J* = 8 Hz, 2H), 3.43 (t, *J* = 8 Hz, 3H), 1.17–1.24 (m, 8H), 0.97 (s, 9H); GC-MS *m*/*z* 199 $[M + H]^+$.

Syn and anti 6-Ethoxycarbonyl-1-methyl-biciclo(3,2,0)hexane, (**7** + **8**). Ethyldiazoacetate, 1.1 mL (13.4 mmol) was added to a solution of 3.9 mL of 1-methylcyclopentene(61.1 mmol) in 15 mL of dry CH_2Cl_2. TPPRh(III)Cl. 20 mg (26 mmol) was added and the solution was refluxed for 24 h under nitrogen. The solution was then evaporated under vacuum and the residue chromatographed on silica gel eluting with hexane/diethyl ether, 99.7:0.3. The first eluted fraction contained 630 mg of the *anti* isomer(yield 30%) while the second fraction contained 470 mg of the *syn* isomer(yield 40%).

Syn isomer:Anal. Calcd for $C_{10}H_{16}O_2$: C, 71.39; H, 9.58; Found: C, 71.65; H, 9.41; ^{1}H-NMR (300 MHz, $CDCl_3$) δ = 4.1 (q, *J* = 12 Hz, 2H), 3.12 (t, *J* = 12 Hz, 3H), 1.9–2.05 (m, 3H), 1.68–1.71 (m, 2H), 1.39–1.45 (m, 2H), 1.18–1.27 (m, 4H); GC-MS *m*/*z* 169 $[M + H]^+$.

Anti isomer:Anal. Calcd for $C_{10}H_{16}O_2$: C, 71.39; H, 9.58; Found: C, 71.50; H, 9.52; ^{1}H-NMR (300 MHz, $CDCl_3$) δ = 4.25 (q, *J* = 12 Hz, 2H), 4.02 (t, *J* = 12 Hz, 3H), 1.65–1.82 (m, 3H), 1.44–1.62 (m, 3H), 1.20–1.30 (m, 5H); GC-MS *m*/*z* 169 $[M + H]^+$.

Cyclopropanation Reactions

In a typical experiment, 0.11 mL (1.34 mmol) of ethyldiazoacetate (EDA) and 0.39 mL (3.7 mmol) of stirene were dissolved in 3 mL of dry $CHCl_3$. 20 mg of catalyst **4** was added and the resulting solution was refluxed for 12 h. An internal standard was added and the reaction mixture was analyzed by GC.
